# Noise-induced anti-correlated slow fluctuations in networks of neural populations

**DOI:** 10.1186/1471-2202-14-S1-P214

**Published:** 2013-07-08

**Authors:** Dongmyeong Lee, Seunghwan Kim, Tae-Wook Ko

**Affiliations:** 1 Department of Physics, Pohang University of Science and Technology, Pohang 790-784, Republic of Korea; 2 Institute for Edge of Theoretical Science, Pohang University of Science and Technology, Pohang 790-784, Republic of Korea; 3 National Institute for Mathematical Sciences, Daejeon 305-811, Republic of Korea

## 

Coherent spontaneous fluctuations (< 0.1Hz) in fMRI blood-oxygen-level-dependent (BOLD) signal have been observed for a resting state of the human brain [1-4]. Functional connectivity analysis has identified clusters of brain areas exhibiting correlated fluctuations [1-4] and anti-correlation relationship between task-positive and task-negative areas [2-4]. In this study, we propose a model explaining the generation of slow fluctuations and the organization of the clusters. Based on the slowness and the anti-correlation relationship, we describe the brain as a network of neural populations which act as brain areas and prefer one of the two states, UP (active) state and DOWN (quiescent) state [5], and consider excitation-inducing or inhibition-inducing connections between brain areas. Without noise, this system can have multiple stable states in which each area can be in UP, DOWN, or intermediate state. Presence of noise can make the system slowly move from one stable state to other and this is manifested as organized slow fluctuations. We implement this mechanism using a Wilson-Cowan model [6,7] with excitatory and inhibitory neurons constituting the neural populations. The neural activity is translated into BOLD signal through the Balloon-Windkessel hemodynamic model [8,9]. With various networks with 2, 3, and 4 nodes, we show that the system without noise can have multiple stable states which are fixed points, and observe slow fluctuations and various organization including anti-correlated clusters. Similar behaviors are observed in the cases with random networks and modular networks. We analyze the functional connectivity in connection with the underlying networks.
